# Genetic Characterization of Hantaviruses Transmitted by the Korean Field Mouse (*Apodemus peninsulae)*, Far East Russia

**DOI:** 10.3201/eid0808.010494

**Published:** 2002-08

**Authors:** Kumari Lokugamage, Hiroaki Kariwa, Daisuke Hayasaka, Bai Zhong Cui, Takuya Iwasaki, Nandadeva Lokugamage, Leonid I. Ivanov, Vladimir I. Volkov, Vladimir A. Demenev, Raisa Slonova, Galina Kompanets, Tatyana Kushnaryova, Takeshi Kurata, Kenji Maeda, Koichi Araki, Tetsuya Mizutani, Kumiko Yoshimatsu, Jiro Arikawa, Ikuo Takashima

**Affiliations:** * Hokkaido University, Sapporo, Japan; † National Institute of Infectious Diseases, Tokyo, Japan; ‡ Plague Control Station, Khabarovsk, Russia; § Far Eastern Medical Association, Khabarovsk, Russia; ¶ Russian Academy of Medical Sciences, Vladivostok, Russia; # Hokkaido University School of Medicine, Sapporo, Japan

**Keywords:** Hantavirus, *Apodemus peninsulae*, hemorrhagic fever with renal syndrome, HFRS, Far East Russia

## Abstract

In an epizootiologic survey of 122 rodents captured in Vladivostok, Russia, antibodies positive for hantavirus were found in *Apodemus peninsulae* (4/70), *A. agrarius* (1/39), and *Clethrionomys rufocanus* (1/8). The hantavirus sequences identified in two seropositive *A. peninsulae* and two patients with hemorrhagic fever with renal syndrome (HFRS) from the Primorye region of Far East Russia were designated as Solovey and Primorye, respectively. The nucleotide sequences of the Solovey, Primorye, and Amur (obtained through GenBank) sequences were closely related (>92% identity). Solovey and Primorye sequences shared 84% nucleotide identity with the prototype Hantaan 76-118. Phylogenetic analysis also indicated a close relationship between Solovey, Primorye, Amur, and other viruses identified in Russia, China, and Korea. Our findings suggest that the Korean field mouse (*A. peninsulae*) is the reservoir for a hantavirus that causes HFRS over a vast area of east Asia, including Far East Russia.

Currently, at least 20 serotypes and genotypes of the *Hantavirus* genus (family: *Bunyaviridae*) have been identified worldwide. Rodents are the natural reservoir for hantaviruses, although one virus strain has been isolated from the house shrew (*Suncus murinus*), an insectivore (1). A unique characteristic of hantaviruses is the close association between the virus type and its natural reservoir (2).

Hantaviruses cause two forms of human disease, hemorrhagic fever with renal syndrome (HFRS), and hantavirus pulmonary syndrome (HPS); human infection occurs after the inhalation of aerosolized rodent excreta. HFRS is manifested as high fever, renal dysfunction, and hemorrhage; HPS is characterized by an acute progressive pulmonary edema and a fatality rate of about 40%. Among the hantaviruses that cause HFRS in Eurasia are *Hantaan virus* (HTNV), *Seoul virus* (SEOV), *Puumala virus* (PUUV), and *Dobrava-Belgrade virus* (DOBV) (3), which are carried by the striped field mouse (*Apodemus agrarius*), Norway rat (*Rattus norvegicus*) and black rat (*R. rattus*), bank vole (*Clethrionomys glareolus*), and yellow-necked field mouse (*A. flavicollis*), respectively. DOBV was also found in *A. agrarius* in Europe (4,5). *Sin Nombre virus* (SNV), *New York virus* (NYV), *Black Creek Canal virus* (BCCV), *Bayou virus* (BAYV), *Andes virus* (ANDV), and other related viruses cause HPS in the New World and are carried by the deer mouse (*Peromyscus maniculatus*), white-footed mouse (*P. leucopus*), cotton rat (*Sigmodon hispidus)*, marsh rice rat (*Oryzomys palustris),* and *Oligoryzomys longicaudatus*, respectively (2,6). Although the known genotypes and serotypes have increased in number with advances in the knowledge of epidemiology and epizootiology of hantavirus infection (2), some still-unidentified hantaviruses carried by specific rodent hosts may exist. HFRS is generally known to be endemic to Far East Russia. However, the genetics of hantaviruses that are pathogenic for humans are not well defined. Reed voles (*Microtus fortis)* in Far East Russia were found to harbor two novel hantaviruses, *Khabarovsk virus* (KHAB) and Vladivostok virus (7,8). Another hantavirus, *Topografov virus* (TOPV), was isolated from brown lemmings (*Lemmus sibiricus*). The correlation between these three viruses and their pathogenicity for humans are not yet known (9).

A recent study reported two novel hantaviruses, designated as Amur (AMR) and Far East (FE), that were identified from HFRS patients in Far East Russia (10). The natural reservoir of AMR genotype seems to be *A. peninsulae*, according to a recent study on nucleotide sequence comparisons by Yashina et al. (11).

In 1999, we carried out an epizootiologic survey in a suburb of Vladivostok, Russia, to determine the characteristics of hantaviruses circulating in Far East Russia and to examine the possibility that *A. peninsulae* is a carrier of pathogenic hantaviruses. We detected antibodies to hantaviruses in *A. peninsulae*, and the viral genome characteristics were extremely similar to the newly identified genotype, AMR (10). Using phylogenetic analysis to characterize the sequences of viruses identified from HFRS patients and *A. peninsulae*, we were able to corroborate the assumption of Yashina et al. (11). We also found that *A. peninsulae*-related viruses are pathogenic for humans and are distributed over a large area of east Asia that includes Far East Russia.

## Materials and Methods

We collected sera and organs from wild rodents captured during 1999. We also collected sera and autopsy materials from HFRS patients in two rural villages in the Primorye region of Russia, located 400 km and 600 km from Vladivostok.

Rodent sera were screened for antibodies to HTNV and PUUV or both by indirect immunofluorescent antibody assay (IFA). Vero E6 cells infected with the Hantaan 76-118 strain of HTNV or the Sotkamo strain of PUUV were used as antigen slides. Diluted sera (1:16 and 1:64) were spotted onto the antigen slides and incubated at 37°C for 1 h. After three washes with phosphate-buffered saline (PBS), protein G-conjugated fluorescein isothiocyanate (FITC) (Zymed Laboratories, Inc., San Francisco, CA) was spotted onto the slides. After incubation at 37°C for 1 h, the slides were washed and observed by fluorescence microscopy. Scattered, granular fluorescence in the cytoplasm of infected Vero E6 cells was considered a positive reaction. Antibodies in HFRS patient sera were detected by the same protocol, except for the substitution of FITC-conjugated antihuman immunoglobulin (Ig) G (ICN Pharmaceuticals, Inc., Aurora, OH).

Total RNA was extracted from lung tissues of seropositive *A. peninsulae* with Isogen (Nippon Gene Co., Ltd., Osaka, Japan), which is based on the acid guanidium-phenol-chloroform technique, according to manufacturer’s instructions. Similarly, total RNA was extracted from lung, liver, kidney, spleen, and brain tissues of HFRS patients. Reverse transcription (RT) was carried out at 42°C for 30 min by using Superscript II and random primer (Gibco-BRL, Rockville, MD). Full-length S segments were amplified with Platinum Taq (Gibco-BRL) and HTNV–full S primer for 30 polymerase chain reaction (PCR) cycles of denaturation at 94°C for 30 s, annealing at 55°C for 30 s, and extension at 68°C for 2 min. Amplification of M segments was identical to that of S segments, except for the use of M genome-specific primers (Table 1). Part of the M segment (232 nucleotides) and the entire S segment (except for the 5´ and 3´ ends) were sequenced with primers specific for HTNV or SEOV or both. Amplification of the partial M segment was achieved only with nested PCR. The PCR-amplified products were separated by using a Rapid Gel Extraction kit (Gibco-BRL) according to the manufacturer’s instructions. Purified DNA fragments were cloned into the PCR 2.1 vector provided in the TA cloning kit (Invitrogen Corporation, Carlsbad, CA). The ligated products were transformed into Top 10 competent cells (Invitrogen Corporation) and purified with a Miniprep kit (QIAGEN GmbH, Hilden, Germany). DNA sequencing was performed with the ABI-PRISM Dye Terminator Sequencing kit (Applied Biosystems, Foster City, CA) and an ABI 373-A genetic analyzer.

**Table 1:** Primers used for reverse transcription-polymerase chain reaction and/or sequencing of S and M genome segments of hantaviruses

Gene	Primer name	Primer sequence (5´–3´)	Position
S segment	M13 Fw	ctggccgtcgttttac	
	PEN 215 S Fw	gaattgaaagacaattggc	215–233
	KPS3^a^	tc(a/c)agcatgaaggc(a/t)gaagagat	592–703
	PEN 780 SFw	acagaggcaggcagctttag	780–799
	PEN 1042 S Fw	gcaggatatgcggaatacaa	1042–1061
	HTNV 1390 S Fw	attgcactattattatcagg	1390–1409
	HTNV Full S	ttctgcagtagtagtag(t)a(g)ctccctaa	
	PEN180 S Rv	ttccctgtctgttaatgctc	180–199
	PEN 585 S Rv	tgggcaaggacacatagaga	585–604
	PEN 946 Rv	atgatggtgactcgatgtct	946–965
	PEN 1160 S Rv	gttgtattcccattgactgt	1160–1179
	HTNV 1493 SRv	cacccacaacggattaactg	1493–1512
	M13 Rv	caggaaacagctatgac	
M segment	HS1^a^	ac(a/c)tgtca(c/a)tttgg(a/t)gaccc	2636–2655
	HS2^a^	tcaca(g/a)gcctttattga(g/t)gt	3072–3091
	HS3^a^	t(t/c)aggaa(ga)aaatg(tc)aactttgc	2715–2736
	HS4^a^	acacc(a/t)gaaccccaggc(a/c)cc	3000–3019
	M13 Fw	ctggccgtcgttttac	
	M13 Rv	caggaaacagctatgac	

^a^Primers designed by Yashina et al.

We used the ClustalX program package (version 1.81; available from: URL: ftp://ftp-igbmc.u-strasbg.fr/pub/ClustalX/) to generate the phylogenetic trees by using the neighbor-joining method with 1000 bootstrap replicates. Hantavirus sequences used in the comparisons were obtained from GenBank. The S and M genome sequences used in this study are listed in Table 2.

**Table 2:** Hantavirus sequences used in this study^a^

Virus type	Strain	Source	Country		Accession nos.		References
			Region	Location	M	S	
HTNV							
	SL/AP61/1999	*Apodemus peninsulae*	Far East Russia	Solovey	AB071185	AB071183	This report
	SL/AP63/1999	*A.peninsulae*	Far East Russia	Solovey	AB071186	AB071184	This report
	PRI/H1/2000	Human	Primorye	Cavalerovo	AB071187	—^b^	This report
	PRI/H2/2000	Human	Primorye	Cavalerovo	AB071188	—	This report
	AMR/680	*A.peninsulae*	Far East Russia	Khabarvosk	AF332571	—	11
	AMR/1166	*A.peninsulae*	Far East Russia	Khabarvosk	AF332569	—	11
	AMR/1169	*A.peninsulae*	Far East Russia	Khabarvosk	AF332570	—	11
	AMR/4234	Human	Far East Russia	Amursk	AF172422	—	10
	AMR/4309	Human	Far East Russia	Amursk	AF172423	—	10
	AMR/4313	Human	Far East Russia	Korphovsky	AF172424	—	10
	H8205	Human	China	—	AB030232	—	—
	HTNV261	—	China	Heilongjiang	—	AF252259	—
	Z10	Human	China	Zhejiang	AB027076	AB027108	12
	Chen4	Human	China	Anhui	—	AB027101	12
	Maaji1	*A*. *agrarius*	Korea	—	—	AF321094	Lee PW ^c^
	Maaji-2	Human	Korea	—	—	AF321095	Lee PW ^c^
	HTN 76-118	*A*. *agrarius*	South Korea	—	M14627	M14626	13,14
	Q32	—	China	Guizhou	—	AB027097	12
	HV114	*A*. *agrarius*	China	Hubei	L08753	AB027110	12,15
	A9	*A*. *agrarius*	China	Jiangsu	AF035831	—	16
	Hojo	Human	South Korea	—	D00376	—	17
	FE/7866	Human	Far East Russia	Razdolnoye	AF172439	—	10
	NC167	*Niviventer confucianus*	China	Anhui	AB027115	AB027523	12
	H3	Human	China	Hubei	—	—	18
	H5	Human	China	Heilongjiang	—	—	18
	A3	*A*. *agrarius*.	China	Zhejiang	AB027055	—	12
	B78	Human	China	Shandong	AB027056	AB027093	12
	Q36	*A*. *agrarius*	China	Guizhou	AB027057	AB027094	12
	Q7	*A*. *agrarius*	China	Guizhou	AB02058	AB027095	12
	Q20	*A*. *agrarius*	China	Guizhou	AB027059	AB027096	12
	Niongxia-A	*A*. *agrarius*	China	Niongxia	AB027060	—	12
	Q10	*A*. *agrarius*	China	Guizhou	AB027062	AB027098	12
	A16	*A*. *agrarius*	China	Sanxi	AB027063	AB027099	12
	Q37	*A*. *agrarius*	China	Guizhou	AB027064	AB027100	12
	Q33	*A*. *agrarius*	China	Guizhou	AB027065	AB027102	12
	Bao9	*A*. *agrarius*	China	Heilongjiang	AB027066	AB027103	12
	Jiang13	*A*. *agrarius*	China	Heilongjiang	AB027067	AB027104	12
	Bao14	*A*. *agrarius*	China	Heilongjiang	AB027068	AB027105	12
	Bao10	*A*. *agrarius*	China	Heilongjiang	AB027069	AB027106	12
	Lee	Human	South Korea	—	D00377	—	17
	62HTNV	—	—	—	AB027070	—	12
	6B	—	—	—	AB027071	—	12
	Vaccine	—	—	—	AB027072	—	12
	H2	—	North Korea	—	AB027073	AB027107	12
	HN26-L	*A*. *agrarius*	China	Hainan	AB027074	—	12
	Luyao	Human	China	Shandong	—	AB027109	12
	B659	Human	China	Shandong	S72339	—	18
	Hu	Human	China	Hubei	AB027077	AB027111	12
	Q83	—	—	Guizhou	AB027078	—	12
	B256	—	—	—	AB027079	AB027112	12
	Thailand	*Bandicota indica*	Thailand	—	L08756	—	—
	Topografov	*Lemmus sibericus*	Far East Russia	Siberia	AJ011647	—	9
SEOV							
	L99	*Rattus losea*	China	Jiangxi	AF035833	AF288299	—
	SR11	*R*. *norvegicus*	Japan	Sapporo	M34882	M34881	19
	Gou3	*R*. *rattus*	China	Zhejiang	AB027521	AB027522	12
	NM39	*R*. *norvegicus*	China	Neimeng	AB027080	—	12
	HB55	Human	China	Henan	AF035832	—	17
	Wan	Human	China	Jiangsu	AB027081	—	12
	J12	Human	China	Jieling	AB027082	—	12
	Henan94	*R*. *norvegicus*	China	Henan	AB027083	—	12
	Shanxi	—	—	—	AB027084	—	12
	HN71-L	*R*. *norvegicus*	China	Hainan	AB027085	—	12
	Guang199	—	—	—	AB027086	—	12
	Beijing-Rn	*R*. *norvegicus*	China	Beijing	AB027087	—	12
	c3	Human	China	Hebei	AB027088	—	12
	Hebei4	*Cricetulus barabensis*	China	Hebei	AB027090	—	12
	SD227	—	China	Shangdong	AB027091	—	12
	SD10	*R*. *norvegicus*	China	Shangdong	AB027092	—	12
	Hbei1	Human	China	Hubei	S72343	—	17
	Seoul	*R*. *norvegicus*	South Korea	—	S47716	—	20
	Tchoupitoulas	*R*. *norvegicus*	North America	—	U00473	—	21
	B-1	*R*. *norvegicus*	Japan	—	X53861	—	22
	Girard Point	*R*. *norvegicus*	North America	—	U00464	—	—
DOBV							
	DOB/SLOV	*A*. *flavicollis*	Slovenia	—	L33685	L41916	23
	DOB/SAA	*A*. *agrarius*	Estonia	—	AJ009774	AJ009773	4
SNV	SNV	*Peromyscus maniculatus*	North America	—	L25783	L25784	24
PUUV	PUU/Sot	*Clethrionomys glareolus*	Finland	—	X61034	—	25
	Kamiiso	*C*. *rufocanus*	Japan	Kamiiso	AB011631	—	8
KHAB	Khabarovsk	*Microtis fortis*	Far East Russia	Khabarvosk	AJ011648	—	9

^a^Abbreviations used: HTNV and HTN, *Haantan virus*; SL, Solovey; PRI, Primorye; AMR, Amur; SEOV, *Seoul virus*; DOB and DOBV, *Dobrava-Belgrade virus*; SLOV, Slovenia; SAA, Saarema; SNV, *Sin Nombre virus*; PUUV, *Puumala virus*; and KHAB, *Khabarovsk virus.* ^b^ —, not reported/not used in this study. ^c^Pers. comm.

Formalin-fixed lung, liver, kidney, and brain tissues from an HFRS patient who died of acute renal failure were observed under light microscopy and subjected to immunohistochemical analysis with monoclonal antibodies against Hantaan virus.

## Results

We carried out the epizootiologic survey on 122 rodents captured in a suburb of Vladivostok; results of serologic screening of rodent sera by IFA are shown in Table 3. Identified rodent species included (70) *A. peninsulae*, (39) *A. agrarius*, (8) *C. rufocanus*, (3) *M. fortis*, and (2) *Tamias sibiricus*. Screening by IFA showed that one *A. agrarius* (2.5%), four *A. peninsulae* (5.7%), and one *C. rufocanus* (12.5%) had antibodies to HTNV or PUUV or both. HTNV-antibody titers ranged from 1:32 to 1:512. All the seropositive rodents, except for *C. rufocanus*, lacked antibody against PUUV (Table 4). Lung tissues from seropositive *A. peninsulae* were subjected to RT-PCR to amplify the virus genomes. Two of the four rodents with high IFA titers to HTNV (1:256 and 1:512) were positive by PCR for both the S and M segments of hantavirus.

**Table 3:** Serologic screening by immunofluorescent antibody assay for *Haantan virus* and *Puumala virus* antibodies in rodents, Vladivostok, Russia^a^

Rodent species	No. of sera tested	Positives by IFA (%)	
		HTNV	PUUV
*Apodemus peninsulae*	70	4(5.7)	0
*A. agrarius*	39	1(2.5)	0
*Clethrionomys rufocanus*	8	1(12.5	1(12.5)
*Microtus fortis*	3	0	0
*Tamias sibiricus*	2	0	0
Total	122	6(4.9)	1(0.8)

^a^Abbreviations used: IFA, immunofluorescent antibody assay; HTNV, *Haantan virus*; PUUV, *Puumala virus*.

**Table 4:** *Haantan virus* and *Puumala virus* antibody titers determined by immunofluorescent antibody assay and polymerase chain reaction results

Species	Sample number	IFA antibody titer		PCR
		HTNV	PUU	
*Apodemus peninsulae*	47	256	<16	-^b^
*A. peninsulae*	61	512	<16	+^c^
*A. peninsulae*	63	256	<16	+
*A. peninsulae*	74	64	<16	-
*A. agrarius*	10	32	<16	NA
*Clethrionomys rufocanus*	32	256	256	ND

^a^Abbreviations used: HTNV, *Haantan virus*; PUUV, *Puumala virus*; IFA, immunofluorescent antibody assay; PCR, polymerase chain reaction; NA, not available; ND, not done. ^b^-, negative. ^c^+, positive.

We obtained the clinical histories of two fatal cases of HFRS in the Primorye region. The patients, who lived in villages 400 km and 600 km from Vladivostok, died 8–13 days after the onset of illness; gastrointestinal bleeding and acute renal failure were the causes of death. Serologic screening showed that both patients were positive for hantaviral antibodies. Antibody titers to HTNV and SEOV were apparently higher than to PUUV. We used lung, liver, kidney, spleen, and brain tissues of these HFRS patients for RT-PCR analysis; the lung and kidney tissues of patient no. 1 and the spleen tissue of patient no. 2 were positive for hantaviral M segment.

**Figure 1:**
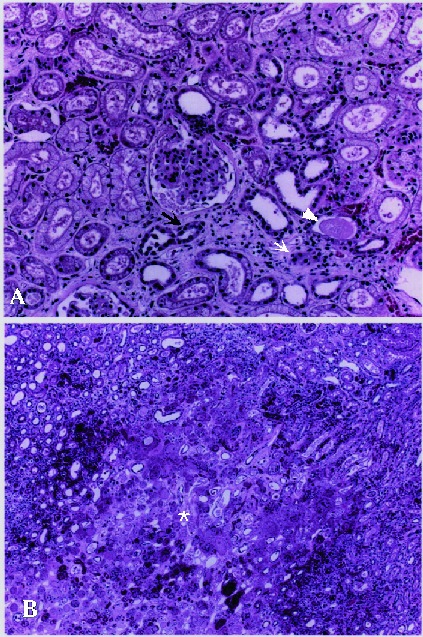
Histopathologic changes in kidney tissue from a patient with hemorrhagic fever with renal syndrome, Primorye region. Changes include interstitial edema with mild infiltration of mononuclear cells (small arrow) and degeneration of renal tubules (large arrow) in cortex. Proteinaceous casts and exudate (arrowhead) are seen in lumina of renal tubules (A). No apparent glomerular changes. Most prominent change in the medulla is well–defined necrotic lesion (asterisk) (B).

To examine the histopathologic changes in HFRS patients, we used light microscopy to examine sections of formalin-fixed lung, liver, kidney, spleen, and brain tissues from patient no. 2, who had died of acute renal failure (Figure 1). The kidney was the only tissue that showed the recognizable histopathologic changes. Salient changes included interstitial edema with mild infiltration of mononuclear cells (Figure 1, small arrow) and degeneration of renal tubules (Figure 1, large arrow) in the cortex (Figure 1, A). Although proteinaceous casts and exudates were observed in the lumina of renal tubules (Figure 1, arrowhead), there were no apparent glomerular changes. In addition, a prominent well-defined necrotic lesion (Figure 1, asterisk) was noted in the medulla (Figure 1, B). Viral antigens were not detected in these specimens by using monoclonal antibodies to HTNV.

The entire S segments of the viruses from two seropositive *A. peninsulae* were amplified and sequenced. We designated these segments as Solovey/AP61/1999 and Solovey/AP63/1999 based on the name of the village closest to the survey point, the rodent species from which the sample was taken, and the year in which the epizootiologic survey was done. We compared the coding regions of these sequences with those of other hantaviruses (Table 5). The S segments of the two Solovey sequences had 99.0% and 98.8% identities in nucleotide and amino acid sequences, respectively. Solovey sequences and Hantaan viruses had 78.2%–84.5% nucleotide sequence identity and 86.7%–93.3% amino acid sequence identity, regardless of their source or geographical origin. Lower nucleotide sequence identities were seen than in Solovey sequences and other viruses: DOBV (73.6%), SEOV (73.9%), and SNV (63.9%).

**Table 5:** Comparison of nucleotide (open reading frame) and amino acid of S genome between those from *Apodemus peninsulae* and other hantaviruses^a^

	Nucleotide and amino acid identities %^b^											
	SL/AP61	SL/AP63	HTNV261	Z10	Chen4	Maaji-1	HTNV 76-118	Q32	NC1167	SR11	GOU3	Dob/Slo
SL/AP61		**99.0**	84.5	83.5	83.4	82.9	82.7	82.3	78.3	73.7	73.7	72.9
SL/AP63	**98.8**		84.2	83.5	83.4	82.9	82.8	81.5	78.2	73.9	73.8	72.2
HTNV261	91.9	91.5		85.6	85.7	83.0	88.6	84.7	78.9	74.1	73.6	72.6
Z10	91.9	91.5	92.9		89.1	83.6	85.9	87.5	79.8	75.3	74.2	73.3
Chen4	93.0	92.5	93.2	96.2		82.8	85.8	90.3	78.7	73.2	74.2	73.4
Maaji-1	91.5	90.8	90.8	91.3	93.0		82.9	82.1	78.2	74.2	73.0	74.2
HTNV76-118	92.2	91.5	94.9	92.9	93.7	91.0		84.4	78.2	74.6	73.8	74.0
Q32	92.7	92.3	93.7	94.4	96.0	91.8	93.2		79.1	73.1	74.3	73.8
NC167	87.2	86.7	85.3	85.8	85.3	84.8	86.9	85.1		75.3	73.6	72.7
SR11	75.0	74.5	74.1	73.9	74.6	74.3	74.8	74.1	77.2		87.8	73.7
GOU3	75.7	75.5	75.0	74.8	76.2	74.3	74.8	76.7	76.7	91.5		73.1
Dob/Slo	76.4	76.4	76.8	75.7	77.6	76.6	75.5	77.2	76.0	73.1	73.1	

^a^Values in bold show the close identities between the two Solovey sequences. Abbreviations used: SL, Solovey; HTNV, Haantan virus; Dob, Dobrova; Slo, Slovenia. ^b^Values above the diagonal and to the right show nucleotide identities; those below the diagonal and to the left show amino acid identities.

To explore the genetic diversity of hantaviruses identified in *A. peninsulae* in more detail, we sequenced the partial M segment of the G2 region (232 nt). We also sequenced the partial M segments of genetic lineages identified in the two HFRS patients from the Primorye region, designated as Primorye/H1/2000 and Primorye/H2/2000. The M segment of Solovey and Primorye sequences were compared with those of other hantaviruses (Table 6). Nucleotide sequence identities among these sequences were between 92.2% and 98.2%; amino acid sequence identities were almost identical (98.7%–100%). We also compared the M segment sequences of Solovey and Primorye with those of AMR genetic lineage, recently identified in HFRS patients and *A. peninsulae* in Far East Russia (10,11). The nucleotide and amino acid identities between Solovey, Primorye , and AMR lineages were 91.3%–98.3% and 93.5%–98.7%, respectively. The M segment sequences of Solovey, Primorye , and AMR lineages were compared with that of H8205, isolated from an HFRS patient in China. In this case, the nucleotide sequence identities were 93.5%–96.1%, and the amino acid sequence identities were 94.8%–100%. Lower nucleotide identities were seen with HTNV (78.8%–86.2%), SEOV (79.3%–81.4%), and DOBV (75.8%–77.1%). This high level of sequence identity among Solovey, Primorye, AMR, and H8205 sequences suggests that some patients acquired the infection from the Korean field mouse (*A. peninsulae*) in Far East Russia and China. Our results also suggest that this genetic lineage is widely distributed throughout east Asia.

**Table 6:** Comparison of nucleotide (bases 2737–2969)^a^ and amino acid of M genome between those from Primorye patients, *Apodemus peninsulae*, and other hantaviruses

	Nucleotide and amino acid identities % ^b^															
	SL/ AP61	SL/ AP63	AMR/ 1169	PRI/ H1	PRI/ H2	H8205	AMR/ 4313	HV 114	A9	HTNV 76-118	Hojo	FE	NC167	DOB/ Slo	SR- 11	PUU
SL/AP61^c^		**99.5**	**97.8**	**96.1**	**98.2**	**94.8**	**94.3**	86.2	85.7	84.4	82.7	82.7	79.3	79.3	79.7	60.3
SL/AP63	**100**		**97.8**	**92.2**	**94.3**	**94.8**	**94.3**	85.7	85.3	84.0	82.3	83.1	78.8	80.1	81.4	60.7
AMR/1169	**94.8**	**94.8**		**96.5**	**98.7**	**95.6**	**95.6**	86.6	86.2	84.9	83.1	81.4	79.7	80.1	79.3	60.3
PRI/H1	**100**	**100**	**94.8**		**96.9**	**93.5**	**92.2**	84.0	83.6	83.1	82.3	80.6	79.3	78.8	79.3	60.3
PRI/H2	**98.7**	**98.7**	**93.5**	**98.7**		**94.8**	**94.3**	85.7	85.3	84.0	82.3	81.4	78.8	79.3	78.8	59.4
H8205	**100**	**100**	**94.8**	**100**	**98.7**		**91.3**	83.6	83.1	85.3	84.9	80.6	77.1	79.3	77.1	60.7
AMR/4313	**98.7**	**98.7**	**93.4**	**98.7**	**97.4**	**98.7**		85.7	85.3	83.6	81.8	82.7	78.0	78.0	78.8	59.9
HV114	93.5	93.5	88.3	93.5	92.2	93.5	92.2		99.5	86.6	84.4	87.9	78.4	75.8	83.1	51.9
A9	93.5	93.5	88.3	93.5	92.2	93.5	92.2	98.9		86.2	84.0	87.5	78.0	75.4	81.8	50.6
HTNV76118	94.8	94.8	89.6	94.8	93.5	94.8	93.5	97.4	96.1		94.6	88.7	79.7	78.4	76.7	59.9
Hojo	94.8	94.8	89.6	94.8	93.5	94.8	93.5	97.4	96.1	100		87.9	78.0	78.8	76.7	51.5
FE	92.2	92.2	87.0	92.2	90.9	92.2	90.9	87.9	87.5	97.4	98.7		75.8	73.7	78.4	59.9
NC167	86.8	86.8	80.5	86.8	85.5	86.8	85.5	89.5	88.2	90.8	90.8	88.2		75.4	77.5	49.3
DOB/Slo	88.3	88.3	83.1	88.3	87.0	88.3	87.0	88.3	87.0	87.0	87.0	84.4	81.6		75.0	59.9
SR11	83.1	83.1	79.2	83.1	81.8	83.1	81.8	83.1	81.8	81.8	81.8	83.1	80.3	80.5		56.0
PUUV	53.2	53.2	53.2	53.2	51.9	53.2	51.9	62.9	62.5	51.9	61.6	53.2	61.6	49.4	61.2	

^a^Based on Haantan 76-118. ^b^Values above the diagonal and the right show nucleotide identities; those below the diagonal and to the left show amino acid identities. ^c^Values in bold show the close identities between those sequences. Abbreviations used: SL, Solovey; AMR, Amur; PRI, Primorye; HTNV, *Haantan virus*; FE, Far East virus; DOB, Dobrova; Slo, Slovenia; PUUV, *Puumala virus*.

**Figure 2:**
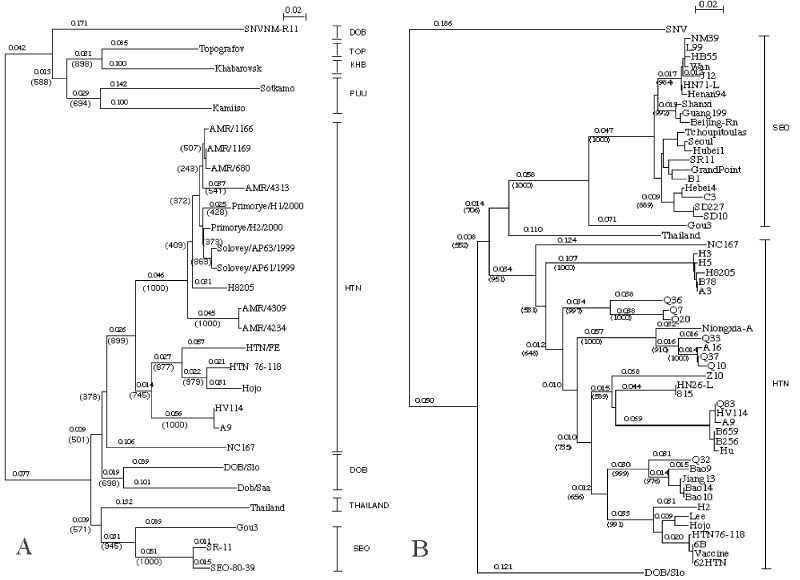
Phylogenetic trees of hantavirus (A) partial M (nt 2736–2968) and (B) partial M (nt 2001–2301) segments. Trees were constructed by using ClustalX (ver. 1.81) program. Numbers above the branches are distances and those in parentheses are bootstrap support values for 1000 replicates.

**Figure 3:**
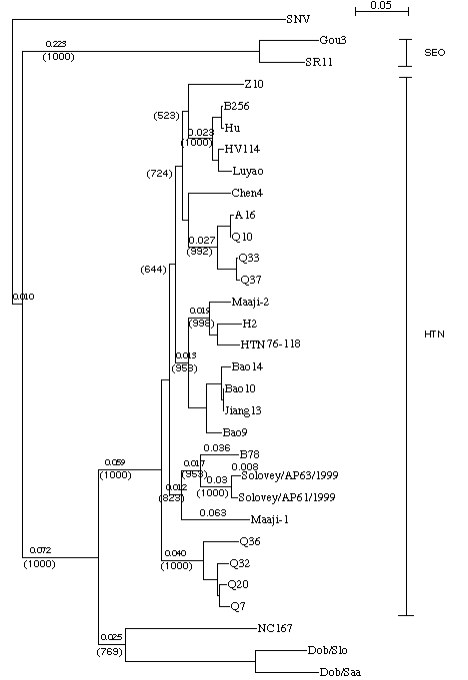
Phylogenetic tree of hantavirus partial S (nt 1216-1666) segments. Tree was constructed by using ClustalX (ver. 1.81) program. Numbers above the branches are distances and in parentheses are bootstrap support values for 1000 replicates.

The M segments of Solovey, Primorye, and AMR sequences formed a common phylogenetic lineage with high bootstrap support values, regardless of viral origin (Figure 2, A). Furthermore, H8205 shared a common lineage with Solovey and Primorye sequences. Another phylogenetic analysis, based on a different region of the M segment, showed that Chinese virus isolates (H8205, H3, H5, and B78) formed a distinct lineage within the Hantaan clade (Figure 2, B). The phylogenetic tree constructed for the S sequences (Figure 3) showed that Solovey sequences formed a single cluster, together with Maaji1 (a Korean isolate) and B78, in a common lineage with high bootstrap support values within the Hantaan clade.

**Figure 4:**
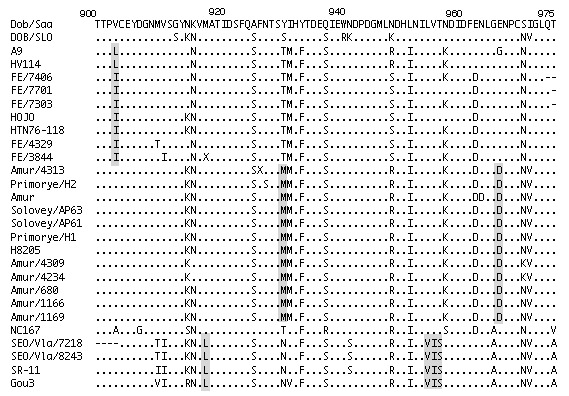
Multiple alignment of partial deduced amino acid sequences of G2 region of hantaviruses. Amino acid sequences analyzed by using ClustalX (ver. 1.8) program. Amino acid positions indicated above sequences based on Haantan 76–118. First line shows the deduced amino acid of Dobrova/Saarema. Dots represent amino acids that are identical to those at corresponding positions in Dobrova/Saarema sequence. Amino acids that differ from those in the sequence are indicated at relevant positions. Hyphens are used in areas where amino acid sequence is not available. Signature amino acids are shaded.

To identify signature amino acids for each virus type, we compared the deduced partial amino acid sequences of their G2 regions using ClustalX multiple-sequence alignment (Figure 4). The presence of leucine or isoleucine at amino acid position (aa) 903 was unique to HTNV except for AMR lineage. The signature amino acids for SEOV were leucine at aa 918 and valine, isoleucine, and serine at aa 955-957. The signature amino acids for AMR lineage were methionine at aa 932 and aspartic acid at aa 967.

## Discussion

 Each hantavirus serotype or genotype is generally associated with a specific rodent host, and various rodent species act as reservoir animals and sources of human infection. Since contact between rodents and humans occurs frequently during agricultural and forestry activities, most infections have been reported in rural areas. However, an urban epidemic of HFRS caused by SEOV has also been reported (26). A large number of rodent species may serve as reservoir animals for pathogenic hantaviruses. For example, few researchers suspected that *P. maniculatus* could transmit highly virulent hantavirus to humans until SNV was identified (27,28). Later studies showed that the other viral agents of HPS such as NYV, BCCV, BAYV, and ANDV, were carried by *P. leucopus,* *S. hispidus* (29), *O. palustris* (30), and *O.* *longicaudatus* (31), respectively. We emphasize the importance of discovering the characteristics of hantaviruses found in endemic areas and identifying the primary hosts.

Although Far East Russia has long been considered an HFRS-endemic area, few reports describe the hantaviral sequences in this region, and information on reservoir animals carrying pathogenic hantaviruses is limited. Our studies therefore focused on determining the genetic characteristics of hantaviruses circulating in this geographic area. We identified *A. peninsulae* as the natural reservoir rodent for a hantavirus pathogenic for humans in Far East Russia. We also identified hantavirus sequences designated as Solovey and Primorye in *A. peninsulae* and HFRS patients, respectively; genetic analysis showed that these sequences were very closely related to each other. This information and the pathological findings from the HFRS case in which Primorye sequence was identified strongly suggest that the virus of Solovey sequence is the causative agent of HFRS. The nucleotide sequence and phylogenetic analysis also showed that Solovey and Primorye sequences were most closely related to AMR and H8205 sequences from patients in Russia and China, but were clearly distinguishable from the prototype of Hantaan virus. Genetic and phylogenetic analysis indicated that Solovey and Primorye sequences were closely related to AMR, Maaji1, H8205, and B78 sequences, viruses derived from distant areas. While Solovey sequences were identified in a suburb of Vladivostok and PRI sequences in two villages 400 km and 600 km from Vladivostok, the H8205 and B78 viruses were derived in China, and Maaji1 was isolated in Korea. *A. peninsulae* is distributed in the same region where *A. agrarius* is prevalent in Korea (PW Lee, pers. comm.). Recently, AMR sequences were found in both HFRS patients and *A. peninsulae* (11). We suggest that some of the viruses circulating in the area of this study cause severe HFRS and are carried by the same host species, *A. peninsulae*. Comparison of the deduced hantaviral amino acid sequences showed that aspartic acid and methionine represented signature amino acids for AMR genetic lineage, regardless of the region in which the virus was identified or its origin (Figure 4). These signature amino acids may be used to distinguish AMR genetic lineage from other hantaviruses. We conclude from our results that *A. peninsulae* carries a hantavirus that is pathogenic for humans. Since *A.* *peninsulae* is widely distributed in Far East Russia, China, Korea, and Japan, this hantavirus and associated cases of HFRS may also be widely distributed.

In the kidney tissue of one HFRS patient (no. 2) from Primorye region, we detected pathologic changes typical of severe HFRS caused by hantavirus infection (32–35). We also detected and sequenced the partial M segment in the spleen of the same patient. However, we could not detect the viral antigen in the kidney samples, possibly because of low levels of the virus in the kidneys of this patient. Nested PCR allowed the amplification of viral M segments from the spleen, but not from kidney, of this patient.

Through epizootiologic, clinical, pathologic, and sequencing studies, we identified a hantavirus carried by *A. peninsulae* as one of the causative agents of HFRS. We think that this information may be helpful in preventing human infections in East Asia. Controversy persists over whether *A. peninsulae* carries a distinct virus type or a subtype of HTNV. A similar question arises with Dobrava/Slovenia and Dobrava/Saaremaa, which are carried by *A. flavicollis* and *A. agrarius*, respectively. The S segment identities between Dobrava/Slovenia and Dobrava/Saaremaa (both obtained from GenBank for comparison purposes) were 87.8% (nucleotide) and 92.7% (amino acid). Similarly, the nucleotide and amino acid sequence identities of the S segments of Solovey sequences and HTN 76-118 were 82.7% and 92.2%, respectively. We suggest that Solovey sequences belong to a sublineage within the HTNV clade.
